# Diagnostic Challenge of Primary Psychiatric Catatonia Versus Seronegative Autoimmune Encephalitis in Pediatrics: A Case Report

**DOI:** 10.7759/cureus.117105

**Published:** 2026-09-28

**Authors:** Sandra Ohabuenyi, Charita Kunta, Kingsley Udom, Neha Bhagi, Delete Ebere-Bank

**Affiliations:** 1 Pediatrics, John H. Stroger, Jr. Hospital of Cook County, Chicago, USA; 2 Pediatrics and Child Health, Rush University, Chicago, USA

**Keywords:** adolescent, catatonia, electroconvulsive therapy, immunotherapy, lorazepam, pulmonary embolism, seronegative autoimmune encephalitis

## Abstract

Catatonia in adolescents presents a diagnostic challenge when autoimmune and psychiatric etiologies overlap. We describe a 16-year-old male with recurrent severe catatonia, complete mutism, and functional decline over four months. Despite a comprehensive negative autoimmune workup, inflammatory markers (elevated interleukin-2 receptor) and clinical features suggested seronegative autoimmune encephalitis. The patient required high-dose lorazepam (18 milligrams per day) and received empiric immunotherapy with intravenous immunoglobulin and corticosteroids, followed by enrollment in a specialized electroconvulsive therapy program. He demonstrated substantial improvement with the return of bilingual speech and social interaction. This case illustrates the importance of maintaining a broad differential diagnosis in adolescent catatonia and considering empiric immunotherapy when clinical suspicion for autoimmune etiology remains high despite negative antibody testing.

## Introduction

Catatonia is a neuropsychiatric syndrome characterized by mutism, stupor, negativism, and decreased initiation that occurs in 0.6% to 17% of psychiatrically hospitalized children and adolescents [1]. Its etiologies span primary psychiatric disorders, autoimmune-mediated conditions, infectious diseases, metabolic disturbances, and toxic exposures [2]. Because the hallmark features manifest identically across these diverse etiologies, distinguishing a primary psychiatric catatonia from catatonia secondary to an underlying medical or neurological process is a genuine and consequential diagnostic challenge in adolescent psychiatry.

Anti-N-methyl-D-aspartate receptor (NMDAR) encephalitis is the most commonly identified autoimmune cause of catatonia in young people, yet seronegative variants present even greater diagnostic complexity [3]. The Graus criteria provide a structured clinical approach to autoimmune encephalitis (AE) when no antibody is detected [4]: “possible” AE requires a compatible subacute clinical presentation with reasonable exclusion of alternative causes, whereas “probable” antibody-negative AE additionally requires objective paraclinical support - cerebrospinal fluid (CSF) pleocytosis, magnetic resonance imaging (MRI) features of encephalitis, or biopsy evidence. This distinction matters therapeutically, because immunotherapy decisions in seronegative cases rest on the strength of this supporting evidence.

We present a case of prolonged severe catatonia in an adolescent male with an extensive negative autoimmune workup and only borderline, nonspecific paraclinical findings. We report it not as a confirmed case of seronegative AE but as an illustration of the diagnostic dilemma that arises when clinical suspicion for an autoimmune process persists despite evidence that falls short of formal diagnostic criteria, and when psychiatric and autoimmune explanations remain in genuine competition.

## Case presentation

A 16-year-old male presented to the emergency department with worsening catatonia reported by his mother. Over the preceding 48 hours, he had developed complete mutism, absence of spontaneous movement, inability to maintain oral intake, and loss of independent ambulation. He remained afebrile and preserved the ability to follow simple commands with vigorous prompting.

His illness had begun approximately two months earlier, following a first-time vaping exposure reported by the family, with decreased oral intake, mutism, and social withdrawal. The vaped substance was never characterized, and urine toxicology screening during the current admission, including cannabinoids, was negative (Table 1). He had been hospitalized at a psychiatric facility for six weeks with partial improvement on lorazepam and amantadine but never returned to baseline. This psychiatric admission and the current presentation represented one continuous illness episode without an interval of recovery; the course is summarized in Table 2. The family history was notable for paternal bipolar disorder.

**Table 1:** Summary of investigations Additional studies (e.g., thyroid function, ammonia, ANA/ENA, ESR/CRP, viral PCR studies) were reported as a comprehensive negative workup; complete itemized results were not fully retrievable at the time of writing and are noted in the limitations. EEG: electroencephalogram; CSF: cerebrospinal fluid; IL-2: interleukin-2; AQP4: aquaporin-4; NMDAR: N-methyl-D-aspartate receptor; MOG: myelin oligodendrocyte glycoprotein; PCP: phencyclidine; ANA: antinuclear antibody; ENA: extractable nuclear antigen; CRP: C-reactive protein; PCR: polymerase chain reaction

Domain	Investigation	Specimen	Result
Neuroimaging	Head CT	-	Normal; no hemorrhage, mass lesion, or hydrocephalus
Neuroimaging	Brain MRI	-	Normal; no demyelination or features of encephalitis
Electrophysiology	EEG (60-minute)	-	Diffuse slowing; no epileptiform activity
CSF	White blood cell count	CSF	5 cells/µL (at upper limit of normal)
CSF	Red blood cell count	CSF	1,000 cells/µL (traumatic tap)
CSF	Protein	CSF	14 mg/dL (within normal range)
CSF	Differential	CSF	67% lymphocytic predominance (of 5 cells/µL)
Immune marker	Soluble IL-2 receptor (sIL-2Rα/CD25)	Serum	3,263 pg/mL - elevated (reference range 532-1,891 pg/mL); nonspecific marker of T-cell activation
Autoimmune	Anti-AQP4 antibody	Serum	Negative; <1.5 U/mL (reference: negative <3.0 U/mL)
Autoimmune	Neuronal surface antibody panel (anti-NMDAR, LGI1, CASPR2, GABA-B, AMPA, DPPX) and anti-MOG	Serum and CSF	Negative
Metabolic	Copper	Serum	<10.0 µg/dL - low
Toxicology	Urine drug screen (cannabinoids, amphetamines, opiates, barbiturates, benzodiazepines, cocaine, methadone, PCP, propoxyphene, fentanyl)	Urine	Negative for all analytes
Toxicology	Alcohol/salicylate	Blood	<10 mg/dL/<2.5 mg/dL (not detected)
Drug level	Acetaminophen	Serum	129 (consistent with therapeutic dosing during admission)

**Table 2:** Chronological clinical timeline IVIG: intravenous immunoglobulin; ECT: electroconvulsive therapy; IV: intravenous; EEG: electroencephalogram; CT: computed tomography; MRI: magnetic resonance imaging; IL-2: interleukin-2; WBC: white blood cell; CSF: cerebrospinal fluid

Timepoint	Event
~Eight weeks before admission	First-time vaping exposure reported by family (substance never characterized; later urine toxicology negative, including cannabinoids); onset of decreased oral intake, mutism, and social withdrawal
Weeks 1-6 of illness	Psychiatric hospitalization (six weeks); treated with lorazepam and amantadine; partial improvement, no return to baseline (same continuous episode)
Day 0	Emergency department presentation with complete mutism, stupor, inability to maintain oral intake, and loss of independent ambulation; admission
Days 1-4	Diagnostic workup: head CT and brain MRI normal; EEG with diffuse slowing, no epileptiform activity; lumbar puncture (borderline WBC, traumatic tap); negative autoimmune and infectious panels; elevated soluble IL-2 receptor (3,263 pg/mL; reference 532-1,891 pg/mL); negative urine toxicology; IV lorazepam started at 2 mg three times daily
Hospital course, first phase	IV lorazepam titration to a maximum of 20 mg three times daily without sedation or respiratory depression; partial but incomplete response
Following lorazepam titration	Empiric immunotherapy: IVIG 2 g/kg total over five days + methylprednisolone 500 mg IV every 12 hours for five days
Subsequent two weeks	Gradual improvement: prompted responses, increasing spontaneous movement, verbal responsiveness, clear bilingual (English/Arabic) speech, social smiling, supported ambulation
Day 17	Bilateral pulmonary emboli diagnosed; anticoagulation with apixaban started
Late hospital course	Transfer to specialized pediatric ECT program for refractory catatonia
Discharge	Substantial but partial improvement; bilingual communication and social interaction restored; 24-hour supervision required; post-ECT outcome unavailable

On examination, the patient was mute and stuporous and required total assistance with all activities of daily living. The presentation met the Diagnostic and Statistical Manual of Mental Disorders, Fifth Edition (DSM-5) criteria for catatonia on the basis of at least three catatonic signs: stupor, mutism, and withdrawal with refusal of oral intake, accompanied by markedly reduced psychomotor initiation. Negativism, posturing, waxy flexibility, rigidity, and echophenomena were not observed during examination. Standardized catatonia rating scores (Bush-Francis Catatonia Rating Scale (BFCRS) or a pediatric catatonia scale) were not collected prospectively; this is acknowledged as a limitation.

Neurological examination includes cranial nerves grossly intact as testable; muscle tone normal; deep tendon reflexes symmetric, without hyperreflexia; plantar responses flexor; no dyskinesias, tremor, or other abnormal movements; coordination and gait not formally testable at presentation owing to stupor. No focal neurological deficits, seizures, autonomic instability, or fluctuating level of consciousness were documented during the admission.

Head computed tomography and brain magnetic resonance imaging were both within normal limits, excluding hemorrhage, mass lesion, hydrocephalus, demyelination, and MRI features of encephalitis. A 60-minute electroencephalogram (EEG) demonstrated diffuse slowing without epileptiform activity, excluding nonconvulsive status epilepticus; diffuse slowing is a nonspecific finding that can accompany metabolic, toxic, medication-related, psychiatric, and systemic states.

CSF analysis showed a white blood cell count of 5 cells per microliter (at the upper limit of normal), red blood cells of 1,000 cells per microliter (consistent with a traumatic tap), protein of 14 milligrams per deciliter (within normal range), and a lymphocytic differential predominance of 67%. Given the very low absolute white cell count and the traumatic tap, this profile does not constitute meaningful evidence of central nervous system inflammation. Comprehensive autoimmune and infectious panels were negative, including anti-aquaporin-4 (anti-AQP4) antibody (<1.5 U/mL; reference: negative <3.0 U/mL); the full workup is summarized in Table 1. Urine toxicology was negative for cannabinoids, amphetamines, opiates, barbiturates, benzodiazepines, cocaine, methadone, phencyclidine, propoxyphene, and fentanyl, with undetectable alcohol (<10 mg/dL) and salicylate (<2.5 mg/dL). Serum copper was low (<10.0 micrograms per deciliter). The key abnormal finding was an elevated soluble interleukin-2 (IL-2) receptor of 3,263 picograms per milliliter (reference range 532-1,891 pg/mL; serum) - a nonspecific marker of T-cell activation.

Intravenous lorazepam was initiated at 2 mg three times daily and titrated during the admission to a maximum of 20 mg three times daily without respiratory depression or excessive sedation - a remarkable tolerance that has been described in severe catatonia. Sedation and respiratory status were monitored throughout. At maximum dosing, the patient showed a partial response: he remained predominantly mute, with persistently reduced spontaneous movement and dependence for oral intake and ambulation.

Given the severity of illness, the incomplete benzodiazepine response, and persistent multidisciplinary concern (psychiatry, neurology, and pediatric hospital medicine) for a possible neuroimmune process despite the negative antibody testing and borderline paraclinical findings, empiric immunomodulatory therapy was undertaken as a therapeutic trial: intravenous immunoglobulin at a total dose of 2 grams per kilogram over five days and pulse corticosteroids (500 mg intravenous methylprednisolone every 12 hours for five days), with standard premedication for infusion-reaction prophylaxis. Because lorazepam, immunotherapy, and intensive supportive care were administered concurrently, the contribution of any single intervention to the subsequent improvement cannot be determined.

Over the subsequent two weeks, the patient showed gradual improvement: emergence of prompted responses, increasing spontaneous movement, beginning verbal responsiveness, and eventually clear verbal output in both English and Arabic. He demonstrated social smiling, laughing, spontaneous question-asking, and ambulation with physical support.

On day 17 of hospitalization, he was diagnosed with bilateral pulmonary emboli, attributed to prolonged immobility, and anticoagulation with apixaban was initiated. He was subsequently transferred to a specialized pediatric electroconvulsive therapy (ECT) program for continued management of refractory catatonia.

At discharge, he had achieved substantial but partial improvement - return of bilingual communication, social interaction, and supported ambulation - while continuing to require 24-hour caregiver supervision and remaining vulnerable to relapse. Post-transfer ECT details (whether ECT was performed, number of sessions, electrode placement, response, and adverse effects) and longitudinal outcomes were not available at the time of writing and are acknowledged as a limitation.

## Discussion

This case illustrates the diagnostic challenge of distinguishing primary psychiatric catatonia from autoimmune-mediated catatonia in adolescents, and the equal danger of over-attributing an undifferentiated catatonic syndrome to an autoimmune process when the supporting evidence is weak.

Assessment against the Graus criteria

Applying the Graus criteria for probable antibody-negative AE [4], this case does not meet the threshold: CSF pleocytosis greater than 5 cells/µL was absent (the count was 5/µL in a traumatic tap), MRI showed no features of encephalitis, and no seizures or epileptiform activity were documented. Features arguing against an autoimmune encephalitic process include the normal MRI, the negative antibody panel in serum and CSF, the absence of definite CSF pleocytosis, and the absence of seizures, focal neurological deficits, movement disorder or dyskinesias, and autonomic dysfunction. Features that prompted clinical concern - the elevated soluble IL-2 receptor, the EEG diffuse slowing, and the CSF lymphocytic differential - are each nonspecific: soluble IL-2 receptor is a marker of T-cell activation seen in numerous inflammatory, infectious, autoimmune, and malignant conditions and cannot independently support a diagnosis of AE; diffuse EEG slowing accompanies metabolic, toxic, medication-related (including high-dose benzodiazepine), psychiatric, and systemic states, as well as severe catatonia itself; and a lymphocytic predominance among only 5 WBC/µL in a traumatic tap carries little weight. The case is therefore best characterized as severe adolescent catatonia with possible autoimmune contribution, at most meeting criteria for “possible” rather than “probable” AE.

Psychiatric differential diagnosis

The psychiatric differential deserves equal emphasis, particularly given the paternal history of bipolar disorder. Catatonia is strongly associated with mood disorders: bipolar disorder with catatonic features is a leading consideration in an adolescent with a first-degree relative with bipolar illness, and catatonia may herald or accompany a first mood episode. Major depressive disorder with catatonic features (depressive stupor) can produce an identical picture of mutism, withdrawal, and refusal of intake. Schizophrenia-spectrum and other psychotic disorders with catatonia remain possible, though no hallucinations, delusions, or formal thought disorder were documented in this patient. Substance-induced catatonia was considered given the temporal association with vaping; however, the exposure was never characterized, urine toxicology, including cannabinoids, was negative on admission, and no causal link can be established. Because the patient’s mutism limited assessment of internal states, mood and psychotic symptoms could not be definitively excluded - a limitation that itself is a reason for diagnostic humility. Moreover, improvement during treatment is equally compatible with the natural course of a primary psychiatric catatonia, and no causal attribution to immunotherapy is possible given concurrent lorazepam, immunotherapy, amantadine (earlier), structured inpatient rehabilitation, and later ECT referral. The temporal association between first-time vaping exposure and symptom onset is noted for completeness; the exposure was never toxicologically characterized, urine toxicology (including cannabinoids) during the admission was negative, and no causal inference can be drawn from a single case, although nicotine exposure has been shown to induce inflammation and modulate adaptive immunity [5].

The patient's tolerance of intravenous lorazepam up to 20 mg three times daily (60 mg/day) without sedation was striking; high benzodiazepine requirements are well described in severe catatonia and should not be taken as evidence of any particular pharmacodynamic mechanism. The decision to trial empiric immunotherapy was an individualized, multidisciplinary judgment in a life-threatening illness with incomplete response to conventional therapy - not a practice recommendation, and not treatment of an established diagnosis. ECT is a well-established treatment for malignant or refractory catatonia, with reported remission rates of approximately 70% to 90% [6,7], and transfer to a specialized pediatric program reflected the severity of illness and partial response to first-line therapies.

Seronegative AE is increasingly recognized as a clinically significant entity, comprising approximately 12% to 50% of clinically suspected cases depending on the cohort [8]. Response to empiric immunotherapy in seronegative cases appears generally favorable, with 50% to 70% of patients demonstrating meaningful clinical improvement [9]. A recent pediatric study found that 95% of probable antibody-negative AE patients achieved good one-year outcomes after immunotherapy, though persistent cognitive and behavioral problems were common [10]. These figures describe cohorts that largely met formal probable criteria - a threshold our case does not meet - and should not be read as validating the diagnosis here.

The subsequent development of bilateral pulmonary emboli highlights the systemic morbidity of severe catatonia and the importance of thromboprophylaxis in immobilized patients [11]. Catatonia-associated immobility increases the risk of deep vein thrombosis and pulmonary embolism through venous stasis, and inflammatory states may further contribute to a hypercoagulable state [12].

Limitations

This report has important limitations: (1) standardized catatonia rating scores (BFCRS or a pediatric scale) were not collected prospectively, precluding objective baseline and longitudinal severity measurement; (2) not all examination and laboratory details could be fully retrieved at the time of writing; (3) multiple therapies were administered concurrently, so improvement cannot be attributed to any single intervention, and the natural course of psychiatric illness is an equally plausible explanation; (4) post-transfer ECT details and longitudinal outcomes are unavailable; and (5) as a single case report, no generalizable treatment recommendations can be drawn. Future reports of pediatric catatonia should include serial standardized catatonia ratings at presentation, after benzodiazepine escalation, after immunotherapy, and at discharge, together with fully itemized autoimmune testing and longitudinal follow-up.

## Conclusions

This case highlights the diagnostic complexity of adolescent catatonia and the importance of considering both psychiatric and autoimmune etiologies in parallel. Although the patient’s findings did not establish seronegative AE - the case falls short of the Graus criteria for probable antibody-negative AE - the severity of presentation and concern for possible neuroimmune involvement led to empiric immunotherapy alongside standard catatonia treatment. Improvement occurred under concurrent therapies and cannot be attributed to any one of them. Clinicians should communicate diagnostic uncertainty transparently to patients and families, remain vigilant for complications of prolonged immobility such as thromboembolism, and prioritize standardized catatonia ratings, detailed autoimmune testing, and longitudinal outcomes in future reports to better clarify diagnosis and treatment response.
